# Bis(2,2′,2′′-nitrilo­triacetamide-κ^3^ *O*,*N*,*O*′)nickel(II) dinitrate tetra­hydrate

**DOI:** 10.1107/S160053681205177X

**Published:** 2013-01-09

**Authors:** Xiao-Hui Deng, Qi-Jun Nie, Feng-Juan Zhu

**Affiliations:** a Institute of Economic Crops, Hubei Academy of Agricultural Science, Wuhan 430064, People’s Republic of China

## Abstract

In the title compound, [Ni(C_6_H_12_N_4_O_3_)_2_](NO_3_)_2_·4H_2_O, the Ni^II^ cation is located on an inversion center and is *N*,*O*,*O*′-chelated by two nitrilo­tris­(acetamide) mol­ecules in a distorted octa­hedral geometry. The complex cations, nitrate anions and lattice water mol­ecules are connected by O—H⋯O and N—H⋯O hydrogen bonds, forming a three-dimensional supra­molecular structure.

## Related literature

For related metal complexes, see: Niraj *et al.* (2012 ▶); Biswajit *et al.* (2009 ▶); Ben Amor *et al.* (1998 ▶). For the synthesis of the ligand, see: Donald & George (1974 ▶).
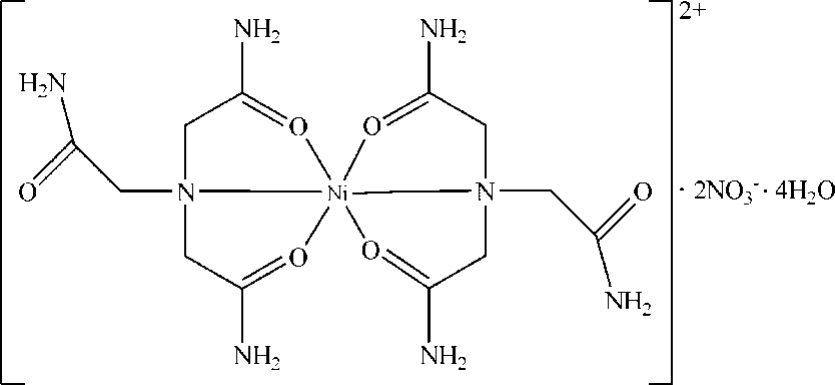


## Experimental

### 

#### Crystal data

[Ni(C_6_H_12_N_4_O_3_)_2_](NO_3_)_2_·4H_2_O
*M* *_r_* = 631.17
Triclinic, 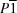

*a* = 8.557 (7) Å
*b* = 9.212 (8) Å
*c* = 9.367 (8) Å
α = 91.180 (14)°
β = 96.215 (14)°
γ = 111.136 (14)°
*V* = 683.2 (10) Å^3^
*Z* = 1
Mo *K*α radiation
μ = 0.80 mm^−1^
*T* = 296 K
0.42 × 0.38 × 0.33 mm

#### Data collection

Bruker SMART 1000 CCD area-detector diffractometer
Absorption correction: multi-scan (*SADABS*; Bruker, 2001 ▶) *T*_min_ = 0.731, *T*_max_ = 0.779
3732 measured reflections
2352 independent reflections
2219 reflections with *I* > 2σ(*I*)
*R*_int_ = 0.014

#### Refinement

*R*[*F* ^2^ > 2σ(*F* ^2^)] = 0.032
*wR*(*F* ^2^) = 0.088
*S* = 1.05
2352 reflections
190 parameters
6 restraints
H atoms treated by a mixture of independent and constrained refinement
Δρ_max_ = 0.31 e Å^−3^
Δρ_min_ = −0.24 e Å^−3^

### 

Data collection: *SMART* (Bruker, 2007 ▶); cell refinement: *SAINT* (Bruker, 2007 ▶); data reduction: *SAINT* ; program(s) used to solve structure: *SHELXTL* (Sheldrick, 2008 ▶); program(s) used to refine structure: *SHELXTL*; molecular graphics: *SHELXTL*; software used to prepare material for publication: *SHELXTL*.

## Supplementary Material

Crystal structure: contains datablock(s) I, New_Global_Publ_Block. DOI: 10.1107/S160053681205177X/xu5668sup1.cif

Structure factors: contains datablock(s) I. DOI: 10.1107/S160053681205177X/xu5668Isup2.hkl

Additional supplementary materials:  crystallographic information; 3D view; checkCIF report

## Figures and Tables

**Table 1:** Hydrogen-bond geometry (Å, °)

*D*—H⋯*A*	*D*—H	H⋯*A*	*D*⋯*A*	*D*—H⋯*A*
N2—H2*A*⋯O8^i^	0.86	2.14	2.988 (3)	169
N2—H2*B*⋯O6^ii^	0.86	2.19	3.027 (4)	165
N3—H3*A*⋯O4^iii^	0.86	2.28	3.056 (4)	150
N3—H3*B*⋯O3^ii^	0.86	1.99	2.848 (3)	173
N4—H4*A*⋯O7^iv^	0.86	2.22	3.002 (3)	152
N4—H4*B*⋯O7	0.86	2.32	3.068 (4)	145
O7—H7*A*⋯O4	0.87 (2)	2.08 (2)	2.913 (4)	162 (3)
O7—H7*B*⋯O8^v^	0.87 (2)	1.98 (2)	2.843 (3)	174 (4)
O8—H8*A*⋯O1^iii^	0.86 (2)	2.18 (2)	3.018 (3)	165 (3)
O8—H8*B*⋯O4	0.86 (2)	2.19 (2)	2.999 (4)	157 (3)
O8—H8*B*⋯O6	0.86 (2)	2.40 (3)	3.107 (4)	141 (3)

Symmetry codes: (i) 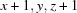; (ii) 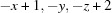; (iii) 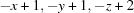; (iv) 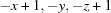; (v) 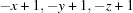.

**Fig. 1.**
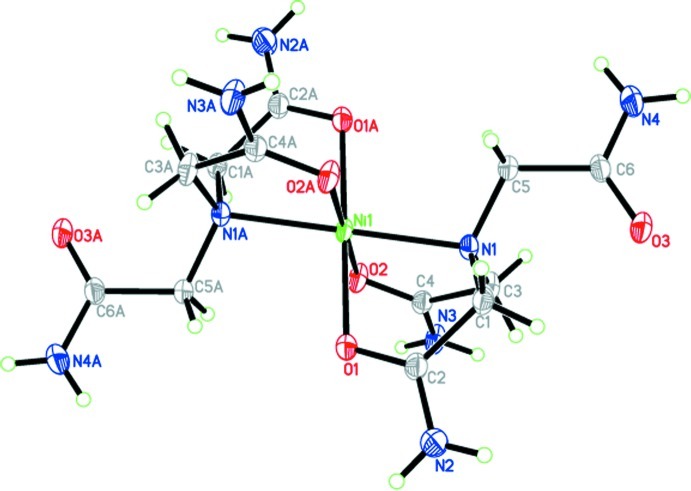
The molecular structure of (I), showing the atom-numbering scheme. Displacement ellipsoids are drawn at the 30% probability level.

**Fig. 2.**
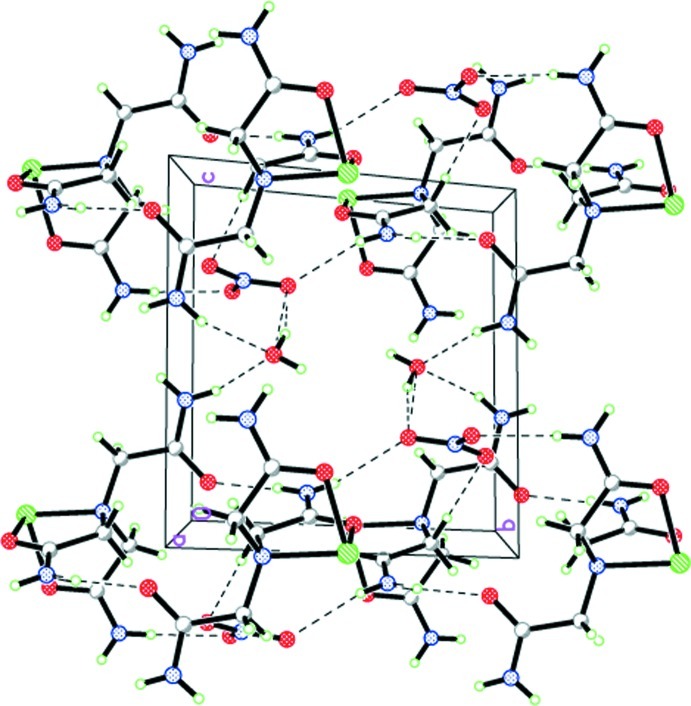
The packed diagram for the title compound, viewed down the a axis with hydrogen bonds drawn as dashed lines.

## supplementary crystallographic information

### Comment

Coordination chemistry of nitrilotriacetic acid with metal ions is explored extensively owing to their flexible coordinating nature, but nitrilotriacetamide (H_3_NTA) is hardly studied (Niraj *et al.*, 2012; Biswajit *et al.*, 2009; Ben Amor *et al.*, 1998). This is the first report of a bis(H_3_NTA)–nickel(II) structure in which only H_3_NTA acts as a tridentate ligand.

Complex I consists of a Ni(H_3_NTA)_2_ cation, two nitrate anions and four solvent water molecules (Scheme). Ni(II) has an octahedral coordination environment which is centrosymmetric as Ni(II) occupies an inversion center. The Ni atom is coordinated in a planar geometry by the nitrilotriacetamide N and O atoms. Two *trans* axial sites of this coordination environment is occupied by O2 and its symmetry related O2' oxygen atoms from ligands(Fig. 1). In the equatorial plane the Ni—N1 distance is 2.131 (2) Å and the Ni—O1 distance is 2.098 (2) Å. The axial Ni—O2 bond is appreciably shortented which is 2.036 (2) Å. A few more selected bond distances and bond angles are presented in Table 1. The molecules are stacked along the *a* axis and display N—H···O and O—H···O hydrogen-bonds interaction (Fig. 2).

### Experimental

The synthesis of nitrilotriacetamide was carried out according to US patent 3799981 (Donald & George, 1974). The title compound was synthesized by adding solid Ni(NO_3_)_2_.6H_2_O (291 mg, 1 mmol) to a solution of ligands (376 mg, 2 mmol) in ethanol/water (2:1, 20 ml), then the mixture was stirred for 2 h at room temperature. The solution was filtered and the filtrate was allowed to stand in air for 1 d, and blue crystals were formed at the bottom of the vessel on slow evaporation of the solvent at room temperature.Yield: 73%.

### Refinement

Water H atoms were located in a difference Fourier map and the positional parameters were refined, U_iso_(H) = 1.5U_eq_(O). Other H atoms were included in calculated positions with C—H = 0.93 or 0.97 and N—H = 0.86 Å, and refined using a riding-model with *U*_iso_(H) = 1.2U_eq_(C,N).

### Crystal data

**Table:**

[Ni(C_6_H_12_N_4_O_3_)_2_](NO_3_)_2_·4H_2_O	*Z* = 1
*M**_r_* = 631.17	*F*(000) = 330
Triclinic, *P*1	*D*_x_ = 1.534 Mg m^−^^3^
Hall symbol: -P 1	Mo *K*α radiation, λ = 0.71073 Å
*a* = 8.557 (7) Å	Cell parameters from 2322 reflections
*b* = 9.212 (8) Å	θ = 2.4–28.2°
*c* = 9.367 (8) Å	µ = 0.80 mm^−^^1^
α = 91.180 (14)°	*T* = 296 K
β = 96.215 (14)°	Block, blue
γ = 111.136 (14)°	0.42 × 0.38 × 0.33 mm
*V* = 683.2 (10) Å^3^	

### Data collection

**Table:**

Bruker SMART 1000 CCD area-detector diffractometer	2352 independent reflections
Radiation source: fine-focus sealed tube	2219 reflections with *I* > 2σ(*I*)
Graphite monochromator	*R*_int_ = 0.014
phi and ω scans	θ_max_ = 25.0°, θ_min_ = 2.2°
Absorption correction: multi-scan (*SADABS*; Bruker, 2001)	*h* = −10→10
*T*_min_ = 0.731, *T*_max_ = 0.779	*k* = −10→10
3732 measured reflections	*l* = −11→9

### Refinement

**Table:**

Refinement on *F*^2^	Primary atom site location: structure-invariant direct methods
Least-squares matrix: full	Secondary atom site location: difference Fourier map
*R*[*F*^2^ > 2σ(*F*^2^)] = 0.032	Hydrogen site location: inferred from neighbouring sites
*wR*(*F*^2^) = 0.088	H atoms treated by a mixture of independent and constrained refinement
*S* = 1.05	*w* = 1/[σ^2^(*F*_o_^2^) + (0.0494*P*)^2^ + 0.3085*P*] where *P* = (*F*_o_^2^ + 2*F*_c_^2^)/3
2352 reflections	(Δ/σ)_max_ < 0.001
190 parameters	Δρ_max_ = 0.31 e Å^−^^3^
6 restraints	Δρ_min_ = −0.24 e Å^−^^3^

### Special details

**Table:**

Experimental. Selected IR data (cm^-1^): 3315 (*s*), 3192 (*s*), 2935(w), 2783(w), 1666(*s*), 1596(*s*), 1276(*m*), 1134(*m*), 997(*m*), 867(*m*), 729(w), 561(*s*).
Geometry. All e.s.d.'s (except the e.s.d. in the dihedral angle between two l.s. planes) are estimated using the full covariance matrix. The cell e.s.d.'s are taken into account individually in the estimation of e.s.d.'s in distances, angles and torsion angles; correlations between e.s.d.'s in cell parameters are only used when they are defined by crystal symmetry. An approximate (isotropic) treatment of cell e.s.d.'s is used for estimating e.s.d.'s involving l.s. planes.
Refinement. Refinement of *F*^2^ against ALL reflections. The weighted *R*-factor *wR* and goodness of fit *S* are based on *F*^2^, conventional *R*-factors *R* are based on *F*, with *F* set to zero for negative *F*^2^. The threshold expression of *F*^2^ > σ(*F*^2^) is used only for calculating *R*-factors(gt) *etc*. and is not relevant to the choice of reflections for refinement. *R*-factors based on *F*^2^ are statistically about twice as large as those based on *F*, and *R*- factors based on ALL data will be even larger.

### Fractional atomic coordinates and isotropic or equivalent isotropic displacement parameters (Å^2^)

**Table:**

	*x*	*y*	*z*	*U*_iso_*/*U*_eq_	
Ni1	1.0000	0.5000	1.0000	0.02392 (14)	
O1	1.0488 (2)	0.43473 (17)	1.20851 (16)	0.0346 (4)	
O3	0.7111 (2)	−0.06864 (18)	0.86966 (18)	0.0445 (4)	
O2	0.78448 (19)	0.51673 (16)	1.05845 (18)	0.0341 (4)	
O4	0.3921 (3)	0.3138 (2)	0.7047 (3)	0.0796 (8)	
O5	0.3643 (3)	0.0742 (3)	0.7446 (3)	0.0766 (7)	
O6	0.1436 (3)	0.1309 (3)	0.6829 (3)	0.0760 (7)	
O8	0.1390 (3)	0.4509 (2)	0.58844 (19)	0.0503 (5)	
H8A	0.070 (4)	0.467 (4)	0.640 (3)	0.075*	
H8B	0.188 (4)	0.396 (4)	0.635 (3)	0.075*	
O7	0.6156 (3)	0.2788 (2)	0.5043 (2)	0.0618 (6)	
H7A	0.563 (4)	0.311 (4)	0.564 (4)	0.093*	
H7B	0.685 (4)	0.361 (3)	0.470 (4)	0.093*	
N4	0.6721 (3)	−0.0015 (2)	0.6398 (2)	0.0488 (6)	
H4A	0.6150	−0.0964	0.6083	0.059*	
H4B	0.6905	0.0722	0.5816	0.059*	
N1	0.8516 (2)	0.25706 (19)	0.97289 (18)	0.0255 (4)	
N3	0.5292 (2)	0.3768 (2)	1.1244 (2)	0.0383 (5)	
H3A	0.5150	0.4598	1.1533	0.046*	
H3B	0.4526	0.2868	1.1308	0.046*	
N2	1.0204 (3)	0.2211 (2)	1.3335 (2)	0.0504 (6)	
H2A	1.0547	0.2765	1.4138	0.060*	
H2B	0.9931	0.1216	1.3325	0.060*	
N5	0.3008 (3)	0.1727 (3)	0.7132 (3)	0.0495 (6)	
C4	0.6690 (3)	0.3874 (2)	1.0699 (2)	0.0278 (4)	
C1	0.9486 (3)	0.1859 (2)	1.0730 (2)	0.0301 (5)	
H1A	1.0441	0.1794	1.0299	0.036*	
H1B	0.8767	0.0813	1.0920	0.036*	
C6	0.7323 (3)	0.0313 (2)	0.7789 (2)	0.0313 (5)	
C2	1.0099 (3)	0.2884 (2)	1.2128 (2)	0.0316 (5)	
C5	0.8390 (3)	0.2040 (2)	0.8193 (2)	0.0322 (5)	
H5A	0.9522	0.2242	0.7956	0.039*	
H5B	0.7918	0.2669	0.7599	0.039*	
C3	0.6822 (3)	0.2338 (2)	1.0189 (3)	0.0346 (5)	
H3C	0.6623	0.1635	1.0963	0.041*	
H3D	0.5953	0.1855	0.9388	0.041*	

### Atomic displacement parameters (Å^2^)

**Table:**

	*U*^11^	*U*^22^	*U*^33^	*U*^12^	*U*^13^	*U*^23^
Ni1	0.0223 (2)	0.0156 (2)	0.0283 (2)	0.00044 (14)	0.00210 (14)	0.00082 (14)
O1	0.0430 (9)	0.0214 (8)	0.0311 (8)	0.0035 (7)	−0.0013 (7)	0.0001 (6)
O3	0.0518 (11)	0.0231 (8)	0.0437 (9)	−0.0027 (7)	0.0006 (8)	0.0012 (7)
O2	0.0284 (8)	0.0193 (7)	0.0515 (9)	0.0033 (6)	0.0110 (7)	0.0018 (6)
O4	0.0941 (18)	0.0344 (11)	0.1007 (18)	0.0035 (11)	0.0446 (15)	−0.0048 (11)
O5	0.0583 (14)	0.0618 (14)	0.109 (2)	0.0227 (12)	0.0036 (13)	0.0220 (13)
O6	0.0538 (13)	0.0572 (13)	0.123 (2)	0.0250 (11)	0.0171 (13)	0.0198 (13)
O8	0.0604 (13)	0.0489 (11)	0.0404 (10)	0.0185 (10)	0.0068 (9)	0.0036 (8)
O7	0.0663 (14)	0.0445 (11)	0.0573 (13)	−0.0015 (10)	0.0122 (10)	−0.0014 (9)
N4	0.0606 (14)	0.0304 (11)	0.0401 (11)	0.0019 (10)	−0.0052 (10)	−0.0068 (9)
N1	0.0240 (9)	0.0187 (8)	0.0296 (9)	0.0033 (7)	0.0013 (7)	−0.0007 (7)
N3	0.0323 (10)	0.0231 (9)	0.0577 (13)	0.0052 (8)	0.0156 (9)	0.0004 (9)
N2	0.0761 (16)	0.0300 (11)	0.0355 (11)	0.0115 (11)	−0.0064 (10)	0.0045 (9)
N5	0.0567 (15)	0.0376 (12)	0.0546 (13)	0.0139 (11)	0.0196 (11)	0.0053 (10)
C4	0.0258 (10)	0.0242 (11)	0.0303 (11)	0.0062 (9)	0.0010 (8)	0.0019 (8)
C1	0.0325 (11)	0.0195 (10)	0.0342 (11)	0.0060 (9)	−0.0007 (9)	0.0019 (8)
C6	0.0305 (11)	0.0234 (11)	0.0358 (11)	0.0050 (9)	0.0040 (9)	−0.0054 (9)
C2	0.0307 (11)	0.0252 (11)	0.0332 (11)	0.0050 (9)	−0.0017 (9)	0.0021 (9)
C5	0.0372 (12)	0.0216 (10)	0.0292 (11)	0.0011 (9)	0.0024 (9)	−0.0001 (8)
C3	0.0247 (11)	0.0211 (10)	0.0529 (14)	0.0017 (9)	0.0079 (10)	−0.0008 (9)

### Geometric parameters (Å, º)

**Table:**

Ni1—O2	2.036 (2)	N1—C5	1.490 (3)
Ni1—O2^i^	2.036 (2)	N1—C3	1.499 (3)
Ni1—O1^i^	2.098 (2)	N1—C1	1.499 (3)
Ni1—O1	2.098 (2)	N3—C4	1.323 (3)
Ni1—N1	2.131 (2)	N3—H3A	0.8600
Ni1—N1^i^	2.131 (2)	N3—H3B	0.8600
O1—C2	1.270 (3)	N2—C2	1.311 (3)
O3—C6	1.244 (3)	N2—H2A	0.8600
O2—C4	1.259 (3)	N2—H2B	0.8600
O4—N5	1.262 (3)	C4—C3	1.530 (3)
O5—N5	1.239 (3)	C1—C2	1.525 (3)
O6—N5	1.256 (3)	C1—H1A	0.9700
O8—H8A	0.856 (17)	C1—H1B	0.9700
O8—H8B	0.859 (17)	C6—C5	1.537 (3)
O7—H7A	0.868 (18)	C5—H5A	0.9700
O7—H7B	0.866 (18)	C5—H5B	0.9700
N4—C6	1.335 (3)	C3—H3C	0.9700
N4—H4A	0.8600	C3—H3D	0.9700
N4—H4B	0.8600		
			
O2—Ni1—O2^i^	180.0	C2—N2—H2B	120.0
O2—Ni1—O1^i^	92.01 (7)	H2A—N2—H2B	120.0
O2^i^—Ni1—O1^i^	87.99 (7)	O5—N5—O6	119.8 (2)
O2—Ni1—O1	87.99 (7)	O5—N5—O4	121.1 (3)
O2^i^—Ni1—O1	92.01 (7)	O6—N5—O4	119.1 (3)
O1^i^—Ni1—O1	180.000 (1)	O2—C4—N3	122.14 (19)
O2—Ni1—N1	83.50 (8)	O2—C4—C3	121.45 (19)
O2^i^—Ni1—N1	96.50 (7)	N3—C4—C3	116.40 (18)
O1^i^—Ni1—N1	99.63 (7)	N1—C1—C2	108.21 (17)
O1—Ni1—N1	80.37 (7)	N1—C1—H1A	110.1
O2—Ni1—N1^i^	96.50 (7)	C2—C1—H1A	110.1
O2^i^—Ni1—N1^i^	83.50 (8)	N1—C1—H1B	110.1
O1^i^—Ni1—N1^i^	80.37 (7)	C2—C1—H1B	110.1
O1—Ni1—N1^i^	99.63 (7)	H1A—C1—H1B	108.4
N1—Ni1—N1^i^	180.0	O3—C6—N4	123.8 (2)
C2—O1—Ni1	112.16 (13)	O3—C6—C5	121.5 (2)
C4—O2—Ni1	114.27 (14)	N4—C6—C5	114.6 (2)
H8A—O8—H8B	109 (2)	O1—C2—N2	122.5 (2)
H7A—O7—H7B	107 (2)	O1—C2—C1	119.24 (18)
C6—N4—H4A	120.0	N2—C2—C1	118.3 (2)
C6—N4—H4B	120.0	N1—C5—C6	115.90 (17)
H4A—N4—H4B	120.0	N1—C5—H5A	108.3
C5—N1—C3	112.27 (17)	C6—C5—H5A	108.3
C5—N1—C1	112.83 (17)	N1—C5—H5B	108.3
C3—N1—C1	111.36 (17)	C6—C5—H5B	108.3
C5—N1—Ni1	108.65 (12)	H5A—C5—H5B	107.4
C3—N1—Ni1	107.87 (12)	N1—C3—C4	112.21 (16)
C1—N1—Ni1	103.32 (13)	N1—C3—H3C	109.2
C4—N3—H3A	120.0	C4—C3—H3C	109.2
C4—N3—H3B	120.0	N1—C3—H3D	109.2
H3A—N3—H3B	120.0	C4—C3—H3D	109.2
C2—N2—H2A	120.0	H3C—C3—H3D	107.9
			
O2—Ni1—O1—C2	100.65 (16)	O1^i^—Ni1—N1—C1	147.65 (13)
O2^i^—Ni1—O1—C2	−79.35 (16)	O1—Ni1—N1—C1	−32.35 (13)
O1^i^—Ni1—O1—C2	177 (100)	N1^i^—Ni1—N1—C1	−136 (100)
N1—Ni1—O1—C2	16.91 (15)	Ni1—O2—C4—N3	171.21 (17)
N1^i^—Ni1—O1—C2	−163.09 (15)	Ni1—O2—C4—C3	−9.8 (3)
O2^i^—Ni1—O2—C4	178 (100)	C5—N1—C1—C2	159.20 (17)
O1^i^—Ni1—O2—C4	106.73 (15)	C3—N1—C1—C2	−73.5 (2)
O1—Ni1—O2—C4	−73.27 (15)	Ni1—N1—C1—C2	42.05 (18)
N1—Ni1—O2—C4	7.27 (15)	Ni1—O1—C2—N2	−175.82 (19)
N1^i^—Ni1—O2—C4	−172.73 (15)	Ni1—O1—C2—C1	4.2 (3)
O2—Ni1—N1—C5	118.55 (14)	N1—C1—C2—O1	−33.3 (3)
O2^i^—Ni1—N1—C5	−61.45 (14)	N1—C1—C2—N2	146.7 (2)
O1^i^—Ni1—N1—C5	27.60 (14)	C3—N1—C5—C6	−59.4 (2)
O1—Ni1—N1—C5	−152.40 (14)	C1—N1—C5—C6	67.4 (2)
N1^i^—Ni1—N1—C5	103 (100)	Ni1—N1—C5—C6	−178.62 (15)
O2—Ni1—N1—C3	−3.40 (13)	O3—C6—C5—N1	−25.7 (3)
O2^i^—Ni1—N1—C3	176.60 (13)	N4—C6—C5—N1	157.0 (2)
O1^i^—Ni1—N1—C3	−94.34 (14)	C5—N1—C3—C4	−119.78 (19)
O1—Ni1—N1—C3	85.66 (14)	C1—N1—C3—C4	112.6 (2)
N1^i^—Ni1—N1—C3	−18 (100)	Ni1—N1—C3—C4	−0.1 (2)
O2—Ni1—N1—C1	−121.40 (14)	O2—C4—C3—N1	6.6 (3)
O2^i^—Ni1—N1—C1	58.60 (14)	N3—C4—C3—N1	−174.31 (19)

Symmetry code: (i) −*x*+2, −*y*+1, −*z*+2.

### Hydrogen-bond geometry (Å, º)

**Table:**

*D*—H···*A*	*D*—H	H···*A*	*D*···*A*	*D*—H···*A*
N2—H2*A*···O8^ii^	0.86	2.14	2.988 (3)	169
N2—H2*B*···O6^iii^	0.86	2.19	3.027 (4)	165
N3—H3*A*···O4^iv^	0.86	2.28	3.056 (4)	150
N3—H3*B*···O3^iii^	0.86	1.99	2.848 (3)	173
N4—H4*A*···O7^v^	0.86	2.22	3.002 (3)	152
N4—H4*B*···O7	0.86	2.32	3.068 (4)	145
O7—H7*A*···O4	0.87 (2)	2.08 (2)	2.913 (4)	162 (3)
O7—H7*B*···O8^vi^	0.87 (2)	1.98 (2)	2.843 (3)	174 (4)
O8—H8*A*···O1^iv^	0.86 (2)	2.18 (2)	3.018 (3)	165 (3)
O8—H8*B*···O4	0.86 (2)	2.19 (2)	2.999 (4)	157 (3)
O8—H8*B*···O6	0.86 (2)	2.40 (3)	3.107 (4)	141 (3)

Symmetry codes: (ii) *x*+1, *y*, *z*+1; (iii) −*x*+1, −*y*, −*z*+2; (iv) −*x*+1, −*y*+1, −*z*+2; (v) −*x*+1, −*y*, −*z*+1; (vi) −*x*+1, −*y*+1, −*z*+1.
